# Glucokinase intrinsically regulates glucose sensing and glucagon secretion in pancreatic alpha cells

**DOI:** 10.1038/s41598-020-76863-z

**Published:** 2020-11-19

**Authors:** Tilo Moede, Barbara Leibiger, Pilar Vaca Sanchez, Elisabetta Daré, Martin Köhler, Thusitha P. Muhandiramlage, Ingo B. Leibiger, Per-Olof Berggren

**Affiliations:** The Rolf Luft Research Center for Diabetes and Endocrinology, Karolinska Institutet, Karolinska Sjukhuset L1:03, 17176 Stockholm, Sweden

**Keywords:** Exocytosis, Cellular imaging, Cell biology, Cell signalling, Metabolism, Metabolic diseases, Diabetes, Endocrine system and metabolic diseases, Diabetes, Pre-diabetes

## Abstract

The secretion of glucagon by pancreatic alpha cells is regulated by a number of external and intrinsic factors. While the electrophysiological processes linking a lowering of glucose concentrations to an increased glucagon release are well characterized, the evidence for the identity and function of the glucose sensor is still incomplete. In the present study we aimed to address two unsolved problems: (1) do individual alpha cells have the intrinsic capability to regulate glucagon secretion by glucose, and (2) is glucokinase the alpha cell glucose sensor in this scenario. Single cell RT-PCR was used to confirm that glucokinase is the main glucose-phosphorylating enzyme expressed in rat pancreatic alpha cells. Modulation of glucokinase activity by pharmacological activators and inhibitors led to a lowering or an increase of the glucose threshold of glucagon release from single alpha cells, measured by TIRF microscopy, respectively. Knockdown of glucokinase expression resulted in a loss of glucose control of glucagon secretion. Taken together this study provides evidence for a crucial role of glucokinase in intrinsic glucose regulation of glucagon release in rat alpha cells.

## Introduction

Secretion of the blood glucose elevating hormone glucagon by pancreatic alpha cells increases rapidly when blood glucose concentration drops. An increase in glucagon leads to stimulation of glucose production by the liver and thereby an increase in blood glucose levels^1,2^. Additionally, glucagon secretion is stimulated by extrinsic signals from the sympathetic and parasympathetic autonomous nervous system^3–5^. Under normo- and hyperglycaemic conditions glucagon secretion is suppressed, probably by a combination of intrinsic, paracrine and juxtacrine mechanisms^6–9^. It has been demonstrated that hypoglycaemia stimulates glucagon secretion by a mechanism involving a decrease of the cytoplasmic ATP/ADP ratio, a slight activation of ATP-sensitive potassium channels (K_ATP_-channels), an increased activity of P/Q-type Ca^2+^-channels, resulting in a Ca^2+^-influx^9,10^. These findings demonstrate the importance of the electrophysiological mechanisms that link glucose metabolism to electrical activity and hormone secretion. Furthermore, a recent publication by Basco et al.^11^, using mice with an alpha cell-specific knockout of glucokinase, demonstrated the crucial role of glucokinase for regulated glucagon secretion in vivo and from intact islets. Honzawa et al.^12^ suggested another glucose-dependent intrinsic mechanism for the regulation of glucagon secretion, where glucose uptake through the sodium-glucose co-transporter SGLT1 can lead to an increase in intracellular Na^+^, thereby promoting glucagon release. This mechanism seems to be especially relevant for the paradoxical hypersecretion of glucagon in type 2 diabetes^12^. While studies so far have demonstrated the ability of alpha cells in intact islets to respond to hypoglycaemic conditions with increased glucagon secretion and the importance of glucokinase in this context, it is not yet clear whether alpha cells are intrinsically able to regulate glucagon secretion and whether glucokinase is the glucose sensor linking glucose metabolism to the regulation of glucagon release. In the present study, we have addressed this question by manipulation of glucokinase activity and expression in purified single alpha cells combined with measurements of glucagon release by total internal reflection fluorescence (TIRF) microscopy.

## Results and discussion

Glucagon secretion is regulated by a multitude of external and intrinsic pathways. The electrophysiological processes linking lowered glucose concentration with an increased glucagon secretion are very well characterized^9,10^, but the evidence for the identity and function of the glucose sensor for an alpha cell intrinsic glucose regulation of glucagon secretion is still incomplete^11,13^. We wanted to strengthen the evidence for glucokinase as the glucose sensor for an alpha cell intrinsic glucagon regulation by performing a set of experiments at the single cell level.

### Pancreatic alpha cells express the neuroendocrine isoform of glucokinase

The first step of glucose metabolism, the phosphorylation of glucose to glucose-6-phosphate, is catalysed, dependent on the cell type, by either hexokinases I-III or glucokinase. Glucokinase is thought to be a hallmark of glucose sensing cells, i.e. pancreatic beta cells, L cells and K cells of the gut as well as glucose-activated or -inhibited neurons^14,15^. While glucokinase expression in alpha cells has been demonstrated already in 1996^13^, this finding remained contentious, since the sample analysed contained only about 87% alpha cells but also about 13% other cell types including beta cells, which would be sufficient to detect glucokinase transcripts. It was therefore necessary to verify the expression of glucokinase at the single cell level in a pure alpha cell preparation. With our sorting protocol we obtained functional rat alpha cells with a high purity (96.6 ± 1.4%)^16^. To test which hexokinase isoforms are expressed in rat pancreatic alpha cells, we analysed the expression of all four hexokinases in single islet cells. In total, 146 dispersed rat islet cells were analysed and identified as alpha, beta, delta and PP cells by detection of glucagon-, insulin-, somatostatin- and pancreatic-polypeptide mRNA, respectively. As expected, cells identified as beta cells were positive for glucokinase and negative for hexokinase I, II and III expression (n = 30). The few detected delta and PP cells, were also negative for hexokinase I, II and III and positive for glucokinase expression (n = 8 for both cell types). Finally and most importantly, all analysed alpha cells (n = 90) were positive for glucokinase (Ct-value mean 25.1 ± SEM 0.2) and negative for hexokinases I-III expression (no fluorescence above background level within 40 cycles of PCR). The functionality of the primers used to amplify hexokinases I–III was confirmed in rat muscle, brain and intestine, where all three isoforms are expressed. Although we did not detect hexokinase I expression in our single rat alpha cells, we cannot exclude a low level of expression below our detection limit. Presence of hexokinase I in mouse alpha cells was shown in Tabula Muris^17^ and discussed in Basco et al.^11^. To verify that rat alpha cells express the neuroendocrine isoform of glucokinase, we isolated RNA from sorted alpha cells and performed RT-PCR using primers located in exon 1 and 7 as described in^18^. The resulting PCR-products were subcloned and sequenced. Sequence analysis revealed that 60% of glucokinase mRNA is encoding the functional B1^15^ neuroendocrine isoform. The remaining 40% encode non-functional variants of the neuroendocrine isoform of glucokinase earlier described as P1 and P2^19^. Our data confirm the findings by Heimberg et al.^13^ and are in agreement with the results of Segerstolpe et al.^20^, which demonstrated, by using single cell RNA-Seq, that glucokinase is the most abundant glucose-phosphorylating enzyme in human pancreatic endocrine cells.

### Analysis of glucagon release from single alpha cells by TIRF microscopy

The regulation of glucose-dependent glucagon release by pancreatic alpha cells has been intensively discussed (for review see^7,21,22^). There have been reports on alpha cell intrinsic mechanisms^23,24^, as well as reports that suggest a beta or delta cell-dependent down-regulation of glucagon release at rising glucose concentrations by paracrine interactions through insulin, somatostatin, Zn^2+^ or GABA^25–31^. Another model suggests the necessity of a juxtacrine interaction between ephrinA ligands from beta cells and signalling through EphA receptors on alpha cells^32^. However, none of these models explain the whole spectrum of responses seen in alpha cells at different glucose concentrations, in different species and under different circumstances, i.e. isolated alpha cells vs. alpha cells in the intact islet. We focused our interest on one particular mechanism of regulation of glucose-dependent glucagon secretion namely stimulation of glucagon release under hypoglycaemic conditions and inhibition at normoglycemic conditions. In rodent islets there is very little release and little change in secretion from beta or delta cells below 4 mM^33^, which makes regulation of glucagon release by paracrine interactions alone unlikely. We therefore hypothesised the presence of an alpha cell intrinsic mechanism to up-regulate glucagon secretion.

**Figure 1:**
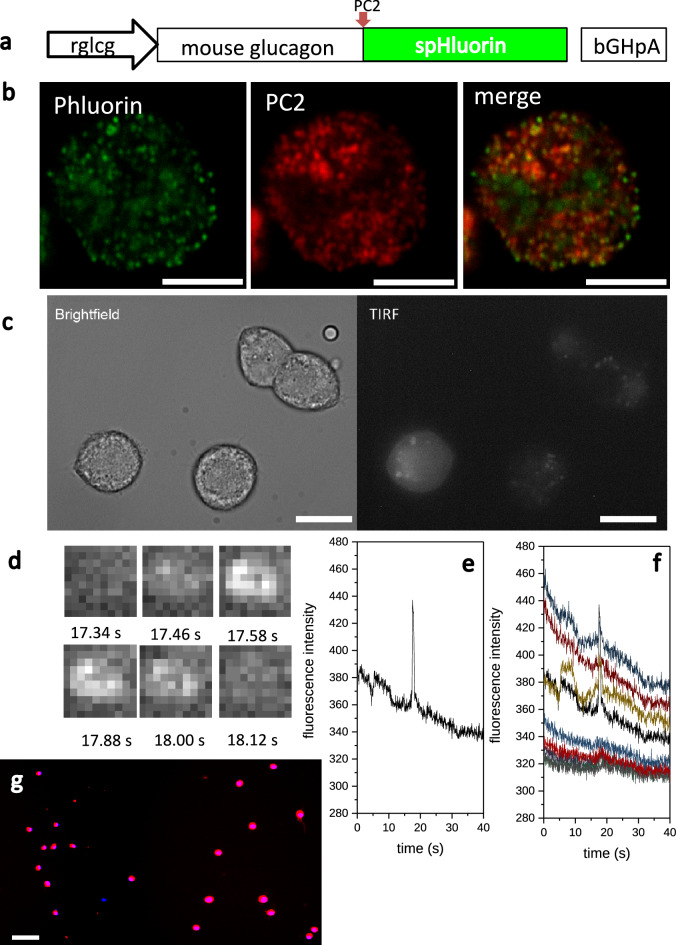
Analysis of glucagon release from single alpha cells by TIRF microscopy. (**a**) Structure of the glucagon release biosensor. The rat glucagon promoter (rglcg, − 776/ + 7 bp) drives the expression of the mouse pro-glucagon (1–69)/spHluorin fusion protein. PC2: position of the prohormone convertase 2 cleavage site, bGHpA: polyadenylation site from bovine growth hormone. (**b**) Confocal image of an alpha cell expressing the biosensor (green) counterstained with PC2 antibody 2 (red), yellow colour in the merged image indicates co-staining for the glucagon release biosensor and PC2 (scale bar 5 µm). (**c**) Brightfield and TIRF image of alpha cells expressing the biosensor (scale bar 10 µm). (**d**) Sequence of 10 × 10-pixel TIRF-images depicting a release event (1pixel = 0.277 µm). (**e**) Fluorescence intensity of the 10 × 10-pixel square, where the depicted release event occurred, over time. (**f**) Fluorescence intensity of the square, where the depicted release event occurred (black line, same as in **e**) and the surrounding 8 squares. (**g**) Immunofluorescence image showing glucagon (red) and DAPI (blue) staining of sorted single rat alpha cells (scale bar 50 µm), observed purity (mean ± SEM) 97.2 ± 0.74% alpha cells, 6 preparations.

To answer the question whether lowering of glucose alone is sufficient to directly stimulate glucagon release and whether glucokinase is acting as the alpha cell glucose sensor, we studied glucose-regulated glucagon release in purified alpha cells to avoid any confounding para- or juxtacrine signals. Our sorting protocol^16^, achieving the highest purity (Fig. 1g), resulted in small amounts of pure alpha cells, which were too few for analyses of glucagon release by either RIA or ELISA. To overcome this problem, we established a technique allowing us to measure glucagon release at the single-cell level. TIRF-microscopy based approaches have been used to successfully study release events in pancreatic beta cells (for review^34^) and with this in mind we adopted this technique to measure glucagon release from alpha cells. We generated a glucagon-pHluorin fluorescent biosensor for glucagon release by replacing the GLP-1- and GLP-2-encoding part within the mouse (prepro)glucagon cDNA with super-ecliptic pHluorin^35^, a pH-sensitive fluorophore that does not fluoresce at acidic pH (Fig. 1a). To achieve selective expression of the construct in primary alpha cells, we used an adenovirus-based vector where the glucagon-pHluorin cassette is driven by the rat glucagon promoter (− 776/ + 7 bp). The pHluorin is co-expressed and co-packaged together with glucagon into secretory vesicles, which was verified by counterstaining with an anti-prohormone convertase 2 (PC2)-antibody. Most vesicles were double positive for pHluorin and PC2, indicating the localization of the biosensor in glucagon-containing granules (Fig. 1b).

**Figure 2:**
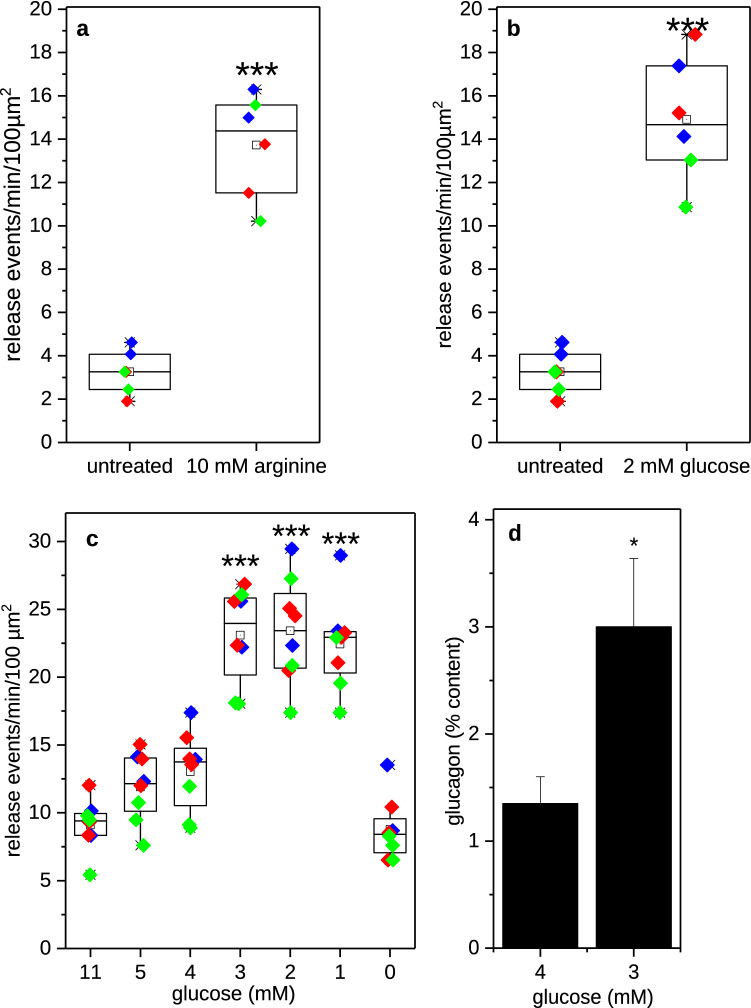
Measurement of glucagon release from sorted alpha cells by TIRF microscopy (**a**–**c**) or by RIA (**d**). (**a**) Single rat alpha cells were imaged for 5 min either at 11 mM glucose (untreated) or 11 mM glucose + 10 mM Arginine (n = 6 cells from 3 preparations, ***p < 0.001 determined by Student’s t-test). (**b**) Single rat alpha cells were imaged for 5 min either at 11 mM glucose (untreated) or 2 mM glucose (n = 6 cells from 3 preparations, ***p < 0.001 determined by Student’s t-test). (**c**) Single rat alpha cells were sequentially exposed to 11, 5, 4, 3, 2, 1 and 0 mM glucose for 3 min each during imaging (n = 8 cells from 3 preparations, ***p < 0.001 vs 11, 5, 4 and 0 mM glucose determined by ANOVA followed by Scheffé Means Comparison). (**a**–**c**) Individual cells are depicted as symbols overlaying the boxplots with different colours indicating different preparation. The raw data can be found in Supplementary Table S1. (**d**) Purified rat alpha cells were incubated for 30 min in either 4 or 3 mM glucose and glucagon release was determined by RIA (n = 5 replicates, *p < 0.05 determined by Student’s t-test, values are mean ± SEM).

Upon fusion of the granules with the plasma membrane, the intragranular pH increases, resulting in a sudden flash of fluorescence of the biosensor that can be recorded as a secretory event. Sorted alpha cells were transduced with the glucagon-pHluorin encoding adenovirus and glucagon release was monitored using a CCD camera at 90 ms/frame recording speed. For analysis the cell surface was divided into 10 × 10 pixel squares and release events were documented as spikes in the fluorescence intensity trace and counted as number of events per 100 µm^2^ and minute (Fig. 1c-f). The functionality of this approach was tested by measuring glucagon release from transduced sorted alpha cells in response to stimulation with 10 mM arginine in the presence of 11 mM glucose (Fig. 2a). A similar secretory response was obtained, when switching from inhibitory high glucose (11 mM) to stimulatory low glucose (2 mM) (Fig. 2b). The latter experiment suggested that low glucose indeed was sufficient to trigger glucagon release from primary rat alpha cells. A similar approach was used recently to study the glucagon release in single human alpha cells^36^, however NPY-EGFP was used to label granules and the loss of fluorescence due to granule emptying was used to identify release events. Nevertheless, the reported number of elicited release events is similar to our observations^36^.

### Low glucose stimulates glucagon release in single rat alpha cells with a threshold comparable to intact islets

Next, we wanted to know more precisely at which glucose concentrations glucagon release is stimulated or inhibited. Therefore, we performed a glucose ramping-down protocol on biosensor-transduced alpha cells starting from 11 mM glucose and lowering glucose concentration in 1 mM-steps from 5 to 0 mM as indicated in Fig. 2c. The results show a two-fold upregulation of glucagon secretion at 1, 2 and 3 mM glucose compared to glucose concentrations of 4 mM or above. This indicated that the threshold for glucagon release from single primary rat alpha cells lies between 4 and 3 mM glucose (Fig. 2c). The release threshold was verified by measuring glucagon secretion from a batch of alpha cells, sorted by the same protocol, using classical glucagon RIA (Fig. 2d). Also this protocol resulted in a two-fold increase of glucagon secretion. We could thereby demonstrate that the change from normoglycemic (4 mM glucose and above) to hypoglycaemic conditions (3 mM glucose or lower) is sufficient to trigger an increase in glucagon release in isolated rat alpha cells. The release threshold is comparable to findings published for intact islets^10,23,37–39^. However, this is in contrast to a report by Reissaus et al.^40^, demonstrating a loss of inhibition of glucagon release by high glucose in isolated mouse alpha cells. Whether this discrepancy is due to species differences or differences in culture conditions remains to be investigated. Interestingly, using glucose-free buffer as a stimulus reduced the number of release events to the level observed at inhibitory glucose concentration. Apparently, the strong reduction of glucose metabolism leads to a further lowering of the ATP/ADP ratio and consequently to both an activation of K_ATP_-channels and inhibition of glucagon release^10^.

### Glucokinase is the glucose sensor for glucose-regulated glucagon release in rat alpha cells

**Figure 3:**
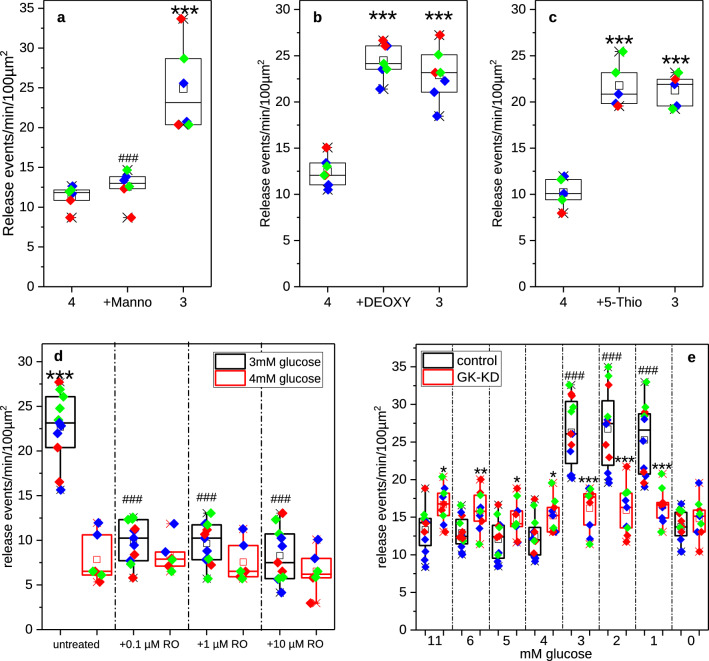
Effect of glucokinase inhibition, activation or knockdown on glucagon release from single rat alpha cells measured by TIRF microscopy. (**a**) Cells were sequentially incubated in 4 mM glucose (4), 4 mM glucose + 10 mM d-Mannoheptulose (+ Manno) or 3 mM glucose (3) (n = 6 cells from 3 preparations; *** p < 0.001 vs 4 mM glucose, ^###^p < 0.001 vs. 3 mM glucose). (**b**) Cells were sequentially incubated in 4 mM glucose (4), 4 mM glucose + 10 mM 2-Deoxy-d-Glucose (+ DEOXY) or 3 mM glucose (3). (n = 7 cells from 3 preparations; ***p < 0.001 vs 4 mM glucose). (**c**) Cells were sequentially incubated in 4 mM glucose (4), 4 mM glucose + 10 mM 5-Thio-d-glucose (+ 5-Thio) or 3 mM glucose (3) (n = 5 cells from 3 preparations; ***p < 0.001 vs 4 mM glucose). (**a–c**) Each incubation lasted for 4 min, with a 4 min washout between inhibitor treatment and 3 mM glucose. (**d**) Cells were incubated in either 4 mM glucose or 3 mM glucose sequentially without (untreated) or rising concentrations (0.1 µM, f or 10 µM) glucokinase activator RO0281675 (n = 6 or n = 11 cells from 3 preparations; ***p < 0.001 vs. untreated 4 mM glucose, ^###^p < 0.001 vs. untreated 3 mM glucose). (**e**) Cells were transfected with negative control (control) or glucokinase siRNA (GK-KD) 5 days prior to the experiment. During imaging, cells were sequentially exposed to each of the glucose concentrations of the ramp (from 11 to 0 mM glucose) for 3 min each. (n = 12 for control or n = 9 for GK-KD cells from 3 preparations; ^###^p < 0.001 vs. other glucose concentration, *p < 0.05, **p < 0.01, ***p < 0.001 for comparison between control and GK-KO). (**a–e**) Statistical significance was determined by one (**a**–**c**) or two (**d**,**e**) way ANOVA followed by Scheffé Means Comparison. Individual cells are depicted as symbols overlaying the boxplots with different colours indicating different preparation. The raw data can be found in Supplementary Table S1.

In order to answer the question whether glucokinase acts as a glucose sensor in glucose-regulated glucagon release, we performed three sets of experiments manipulating the activity or expression of alpha cell glucokinase. In the first experiment, we studied the effect of the classic glucokinase inhibitor mannoheptulose, which turned out not to be a useful tool to investigate the role of glucokinase in alpha cells. Incubation of alpha cells with 10 mM mannoheptulose at 4 mM glucose did not result in an increase of release events (Fig. 3a), which can be explained by the fact that rat alpha cells do not express glucose transporter 2^41^, required for the uptake of mannoheptulose. We therefore used inhibitors of glycolysis to get an indication of the importance of the first steps of glucose metabolism. We used 2-Deoxy-d-glucose, which inhibits glucose-6-phosphate isomerase^42^, and 5-Thio-d-glucose, which has been described as both an inhibitor of glucose transport^43^ and of glucose-6-phosphate isomerase^44^. Incubation of alpha cells with either 10 mM 2-Deoxy-d-glucose (Fig. 3b) or 10 mM 5-Thio-d-glucose (Fig. 3c) at 4 mM glucose led to an increase in number of release events similar to that obtained at stimulatory 3 mM glucose alone. Both inhibitors lower the rate of glucose metabolism^42–44^, which would explain that normally inhibitory 4 mM glucose acts as a stimulator of glucagon secretion.

In the next set of experiments we manipulated glucokinase activity by the glucokinase activator (GKA) RO0281675^45,46^. Here, we studied the effect of different concentrations of GKA on release events at non-stimulatory 4 mM glucose or stimulatory 3 mM glucose. While incubation with GKA at 4 mM glucose had no effect on glucagon release at any GKA concentration up to 10 µM, the stimulatory effect of 3 mM glucose on the number of glucagon release events was inhibited already by the addition of 0.1 µM GKA (Fig. 3d). It has been shown that activation of glucokinase with small molecule glucokinase activators leads to an increased glucose phosphorylation and ATP production even at lower glucose concentration^47^. This can explain that in the presence of glucokinase activators already 3 mM glucose are sufficient to inhibit glucagon secretion.

In the last set of experiments, we tested the effect of knockdown of glucokinase expression in rat alpha cells on glucose-regulated glucagon release. Therefore, sorted alpha cells were first transfected with siRNA against glucokinase (functionality of which was tested in INS-1 cells, Supplementary Fig. S1) and then transduced with the biosensor-encoding adenovirus. After performing the glucagon release experiment, cells were lysed and glucokinase mRNA levels were determined by RT-PCR. The siRNA treatment resulted in reduction of glucokinase expression to 23.5 ± 3.5% (mean ± SEM, n = 3 preparations).

As shown in Fig. 3e, glucokinase knockdown led to a small but significant increase of glucagon release at inhibitory glucose concentrations (between 11 and 4 mM glucose), a reduction of glucagon secretion at stimulatory glucose concentrations (3, 2 and 1 mM glucose) and resulted in complete loss of glucose regulation of glucagon secretion with no significant differences in the amounts of glucagon secreted between the different glucose concentrations applied. This loss of glucose regulation of glucagon secretion due to glucokinase knockdown is in agreement with findings by Basco et al.^11^, which demonstrate loss of glucose regulation of glucagon secretion in mice lacking glucokinase in alpha cells in isolated islets as well as in vivo. The reduction of glucokinase levels in these mice did not lead to a total loss of glucose metabolism, ATP production and glucagon secretion, but to a sufficiently large reduction of metabolic flux to disturb the glucose regulation of glucagon release^11^. The cytoplasmic ATP/ADP ratio is relatively low under glucokinase knockout conditions leading to only small changes in glucagon secretion at different glucose concentrations^11^. We observed an uncontrolled release in glucokinase knockdown cells that is lower than the release observed at low (1–3 mM) glucose but significantly higher than the release observed under normo- or hyperglycaemic conditions in control cells. Basco et al.^11^ report an uncontrolled release under knockout conditions that is at the same level as that observed at low (1 mM) glucose in control islets. This discrepancy may be explained by the different setup of experiments—mouse islets vs. purified single rat alpha cells and static incubation vs. perifusion.

Taken together our findings demonstrate that glucokinase is indeed the intrinsic glucose sensor of the pancreatic alpha cell, linking glucose uptake and glucose metabolism with the electrophysiological machinery described in the literature^7,9,21^ and regulating glucagon release.

## Conclusion

Our work confirmed that pancreatic alpha cells, as other glucose-sensitive cells, express the neuroendocrine form of glucokinase as the most abundant glucose-phosphorylating enzyme. Alpha cells thus function as other glucose-sensing cells equipped with glucokinase. The electrophysiological machinery converts changes in the ATP/ADP ratio into electrical activity and in turn into release events as discussed elsewhere^15,48^. We demonstrate that glucokinase is the glucose sensor, linking glucose uptake and glucose metabolism with glucagon release. Under normal circumstances, lowering glucose to a hypoglycaemic glucose concentration of 3 mM increasees glucagon release. Inhibition of glycolysis, by for example 2-Deoxy-d-glucose or 5-Thio-d-glucose, leads to a shift of the release threshold to higher glucose concentrations while activation of glucokinase lowers the glucose concentration at which release is inhibited. Finally, knockdown of glucokinase leads to a total loss of glucose regulation of glucagon secretion.

While we demonstrate an alpha cell intrinsic mechanism of glucose-regulated glucagon secretion involving glucokinase as the central glucose sensor for the stimulation of glucagon secretion at hypoglycaemic glucose concentrations, we by no means exclude the important role for para- or juxtacrine signalling in the process of inhibiting glucagon secretion at higher, i.e. hyperglycaemic conditions.

## Materials and methods

### Isolation of islets of Langerhans from Wistar rats

The study was performed in accordance with the NIH Guide for the Care and Use of Laboratory Animals and all procedures were approved by the Animal Ethics Committee of Northern Stockholm. Wistar rats (6–10 week-old) were obtained from Scanbur AB (Sollentuna, Sweden). Animals were housed at a constant room temperature (22 °C; 12 h light/dark cycle) with ad libitum access to food and water. The isolation of islets was performed as previously described^16^. Rats were anesthetized with isoflurane (Baxter, Kista, Sweden) before decapitation. Ice-cold isolation buffer (HBSS with 0.5% BSA, 100 units/ml penicillin G, 100 μg/ml streptomycin sulphate and 25 mM HEPES pH 7.4) containing 1 mg/ml collagenase A (Roche, Stockholm, Sweden) was injected into the pancreata. The pancreata were transferred to vials and digested at 37 °C. Then the tissue was washed with isolation buffer and islets were purified using a discontinuous Histopaque density gradient. The islets were washed and hand-picked into complete S-Minimum Essential Medium (S-MEM) (Thermo Fisher Scientific, Waltham, MA, USA), supplemented with 10% FBS, 2 mM glutamine, 100 units/ml penicillin G, 100 μg/ml streptomycin sulphate and 25 mM HEPES.

### Separation of pancreatic alpha cells by flow cytometry

Alpha cells were purified from dissociated islet cells using fluorescence-activated cell sorting (FACS) based on endogenous fluorescence and light scatter. We have previously established a one-step gating strategy that results in functional alpha cells with high purity (96.6 ± 1.4%)^16^. For each FACS preparation islets obtained from 3 to 4 rats were dispersed into single cells by incubation with Accutase (Innovative Cell Technologies, Cytotech, Hellebaek, Denmark). The cells were transferred to sorting buffer (HBSS containing 2.5 mM glucose, 1% BSA, 100 units/ml penicillin G, 100 μg/ml streptomycin sulphate and 25 mM HEPES pH 7.4) and sorted using an Influx cell sorter (BD Biosciences, San Jose, CA, USA) equipped with solid state lasers for 355 nm and 488 nm excitation and a 70 µm nozzle. Forward scatter with parallel polarization (FSC) and side scatter (SSC) were collected at 488 nm. FAD and NAD(P)H types of cellular autofluorescence were excited by 488 nm and 355 nm and collected after 528/38 and 460/50 band-pass filters, respectively. The purified alpha cells were cultured on poly-l-ornithine coated surfaces of either 96 well plates (3000 cells/well) or glass coverslips (~ 1000 cells/coverslip) in complete Improved MEM Zn^2+^ Option (Richter's Modification) medium (Thermo Fisher Scientific, Waltham, MA, USA), supplemented with 10% fetal bovine serum, 100 units/ml penicillin G, 100 μg/ml streptomycin sulphate and 10 mM HEPES pH 7.4. Cells were maintained at 37 °C in a humidified atmosphere with 5% CO_2_.

### Evaluation of glucagon secretion by static incubation

Sorted alpha cells cultured in 96-well plates (3000 cells/well) were washed twice with a buffer containing 0.1% BSA, 125 mM NaCl, 5.9 mM KCl, 1.28 mM CaCl_2_, 1.2 mM MgCl_2_, 5.5 mM glucose and 25 mM HEPES pH 7.4. Cells were incubated in the same buffer for 40 min at 37 °C. After removal, the buffer was replaced by buffer containing different concentrations of glucose, as specified in the results section. After 30 min incubation at 37 °C, the buffer was collected and centrifuged at 4 °C to remove cellular debris. The cells were lysed in the wells with M-PER Mammalian Protein Extraction Reagent (Thermo Scientific, VWR International AB, Stockholm, Sweden). The Glucagon RIA kit GL-32 K from Millipore (Solna, Sweden) was used for determining both the glucagon concentration in the buffer (secreted hormone) and the total cellular glucagon content. Data were analysed using the GraphPad Prism 5 (GraphPad Software Inc., San Diego, USA) with Student’s *t* test. Differences were considered statistically significant at p ≤ 0.05.

### Biosensor construction

Super-ecliptic pHluorin spH^35^ was generated by site-directed mutagenesis employing the QuikChange XL mutagenesis kit (Agilent Technologies, Santa Clara, CA, USA) and respective DNA oligonucleotides (Sigma-Aldrich Sweden AB, Stockholm, Sweden). Introducing the following mutations into the cDNA of enhanced GFP resulted in the generation of pB.0spH: M1K, S147D, N149Q, S202F and Q204T.

Mouse (prepro)glucagon cDNA was generated by RT-PCR using primers MMGCG1 TGTCTACACCTGTTCGCAGC (upstream primer) and MMGCG2 GTGACTGGCACGAGATGTTG (downstream primer) and RNA of glucagon-producing αTC1-9 cells (American Type Culture Collection, Manassas, VA, USA). The cDNA was subcloned into pCRII (Thermo Fisher Scientific, Waltham, MA, USA) generating pCRII.MMGCG. To generate pENTR.rGlcg.MMGCG, we first subcloned the rGlcg.DsRed2 cassette from pGlcg.DsRed2^49^ into pENTR1A (Thermo Fisher Scientific, Waltham, MA, USA) and then exchanged the DsRed2 sequence by the MMGCG cDNA, thus obtaining pENTR.rGlcg.MMGCG. To construct pENTR.rGlcg.MMGCG(1-104)-spH, we first introduced a Cla1-site in the MMGCG sequence thus introducing mutations SD105,106ID and then cloned in-frame the spH cDNA from pB.0spH. All constructs were verified by DNA sequence analysis.

The rGlcg.MMGCG(1-104)-spH-cassette was transferred into the promoterless adenovirus plasmid pAd/PL-DEST (Thermo Fisher Scientific, Waltham, MA, USA) by the Gateway technique. The ViraPower Adenoviral Expression System (Thermo Fisher Scientific, Waltham, MA, USA) was used to generate a replication-deficient adenovirus, which was used for transduction of cells and islets.

### Immunofluorescence

#### Verification of the biosensor by immunofluorescence

Isolated primary rat alpha cells were prepared and transduced as described below. 72 h after start of transductions the cells were fixed with 4% paraformaldehyde for 30 min. They were washed with PBS and incubated with primary antibodies against pro-hormone convertase 2 (PC2, rabbit monoclonal, 1:300, Cell Signalling, Danvers, MA, USA) and GFP (chicken, 1:1000, ABCAM, Cambridge, UK) in the presence of 0.1% Triton-X100 for permeabilisation and 2% BSA for blocking 24 h at room temperature. Cells were washed 3 times with PBS and incubated with a secondary Alexa546-labelled anti-rabbit antibody (1:1000, Thermo Fisher Scientific, Waltham, MA, USA) and a secondary Alexa488-labelled anti-chicken antibody (1:1000, Thermo Fisher Scientific, Waltham, MA, USA) under the same conditions. Imaging was performed using a LEICA SP2 confocal microscope equipped with a 63 × 1.2 NA lens with the following settings: between lines sequential scanning to avoid spectral bleed through, 488/546 double dichroic mirror, Alexa488 excitation at 488 nm, detection at 505–535 nm; Alexa 546 excitation 546 nm, detection 560–620 nm. Image preparation for publication was performed using FIJI^50^.

#### Immunofluorescence of sorted cells

Cells were fixed with 4% paraformaldehyde for 15 min and stained according to a procedure previously described^51^ using mouse monoclonal anti-glucagon antibody (Sigma-Aldrich Sweden AB, Stockholm, Sweden) and secondary goat anti-mouse IgG–Alexa 647 polyclonal antibody (Invitrogen, Stockholm, Sweden). Cells were covered with Vectashield mounting medium containing 1.5 µg/ml 4′,6-diamidino-2-phenylindole (DAPI) (Vector Laboratories, Immunkemi F&D AB, Järfalla, Sweden) and examined with a BD Pathway 855 High-Content Bioimager (BD Biosciences, Rockville, MD, USA) with an Olympus UPlanSApo 10×/0.40 objective. Segmentation of cells based on nucleic DAPI fluorescence staining and subsequent immunofluorescence intensity analysis was performed with the BD Attovision software. Classification and counting of cells was done with the FlowJo Software (Tree Star Inc., Ashland, OR, USA).

### Analysis of glucagon secretion by TIRF microscopy

Cells were maintained in complete Improved MEM Zn^2+^ Option (Richter's Modification) medium, supplemented with 10% fetal bovine serum, 100 units/ml penicillin G, 100 μg/ml streptomycin sulphate and 10 mM HEPES pH 7.4. Sorted alpha cells were seeded onto 25 mm glass coverslips and transduced 24 h later with the biosensor by incubation with 10^7^ pfu/ml of the adenovirus for 4 h. Transduction was performed 72 h prior to the experiments. For imaging experiments the coverslips were transferred to a perifusion chamber and perifused with a buffer containing 0.1% BSA, 125 mM NaCl, 5.9 mM KCl, 1.28 mM CaCl_2_, 1.2 mM MgCl_2_, 25 mM HEPES pH 7.4 and between 1 and 11 mM glucose. TIRF imaging was performed using a ZEISS Axiovert 200 M microscope equipped with a Plan-Fluar × 100/1.45 oil TIRF objective, a TIRF-slider, a LASOS 77 laser for excitation and an AxioCamHS camera for image acquisition. For detection of pHluorin fluorescence the following filter set was used: excitation 488/10 nm, dichroic 492 nm and emission 520/35 nm. Image capture was performed as fast as possible using the camera in 4 × binned mode with an exposure time of 90 ms. Image analysis to identify release events was performed using FIJI^50^. Release events were identified by analysing fluorescence intensity over time in a grid of 10 × 10 pixel squares that were placed over the analysed cell. A release event was detected as peak of fluorescence intensity. If the peak of fluorescence intensity in one square was at the exact same time point but lower than the peak in one of the eight neighbouring squares, it was considered a spillover from a neighbouring square and not counted as a release event. The number of release events was normalized to the surface area of the analysed cell.

Statistical analysis was performed using Excel and Student’s t-test for determination of significance for single comparisons. For multiple comparisons Origin (OriginLab Corporation, Northampton, MA, USA) and ANOVA followed by Scheffé-test for means comparison were used to determine significance levels.

### Pharmacological manipulation of glucokinase activity in sorted alpha cells

Glucokinase activity was manipulated by acute treatment with either 0.1 µM, 1 µM or 10 µM glucokinase activator RO0281675 (Roche) or with 10 mM of the glycolysis inhibitors 5-Thio-d-glucose, 2-Deoxy-d-glucose or d-Mannoheptulose (Sigma-Aldrich Sweden AB, Stockholm, Sweden). The inhibitors were added to medium containing 4 mM glucose, each incubation lasted for 4 min, with a 4 min washout between inhibitor treatment and 3 mM glucose. The glucokinase activator was used at 3 mM and 4 mM glucose. The cells were incubated in either 4 mM glucose or 3 mM glucose sequentially without (untreated) or rising concentrations (0.1 µM, 1 µM or 10 µM) of the glucokinase activator RO0281675.

### Manipulation of glucokinase expression in sorted alpha cells by siRNA mediated glucokinase knockdown

siRNA against rat glucokinase (SI01515878, performance of which was tested in INS-1 cells) and validated non-targeting negative control siRNA (SI1027310) were purchased from QIAGEN (QIAGEN, Hilden, Germany). The sorted cells were transfected first overnight with the siRNA using Lipofectamine 2000 (Thermo Fisher Scientific, Waltham, MA, USA) and 48 h after start of transfection the cells were transduced with the biosensor for 4 h. All experiments were performed 5 days after start of transfection with the siRNA. After the imaging experiment, cells were lysed and knockdown of glucokinase was confirmed by RT-PCR by using glucokinase and Cyclophilin A TaqMan assays (Rn00561265_m1 and Rn00690933_m1) without pre-amplification. Knockdown to 23.5 ± 3.5% (mean ± SEM) was achieved (n = 3).

### Analysis of glucokinase transcripts

RT-PCR, cloning and analysis of glucokinase transcripts were performed as described in^18^. Briefly, mRNA was isolated from FACS-sorted alpha cells using the RNeasy Micro kit (Qiagen, Hilden, Germany). cDNA was synthesized using the SuperScript VILO cDNA Synthesis Kit (Thermo Fisher Scientific, Waltham, MA, USA). The following primers were used: GK-3 5′-AATCTTGCGGAACACTGAG-3′ upstream of the AUG start codon of the neuroendocrine glucokinase and the antisense primer GK-2 5′-CCACATTCTGCATTTCCTC -3′, binding in exon 7^18^. The resulting PCR-products were subcloned into the pCRII vector using the TA Cloning kit (Thermo Fisher Scientific) according to the manufacturors instructions. In total 76 clones were analysed by restriction enzyme digestion and sequencing.

### Single cell RT-PCR

Single cell RT-PCR was performed as described in^52^. Cell types were determined by the detection of insulin-, glucagon-, somatostatin- and pancreatic-polypeptide-mRNA respectively. cDNAs were then pre-amplified using the TaqMan PreAmp Master Mix Kit (Thermo Fisher Scientific, Waltham, MA, USA) and hexokinase I-III and glucokinase (hexokinase IV) expression levels were determined. All expression RNA detection levels were normalized to cyclophilin A (Ppia). The following TaqMan assays were used:

**Table:** 

Gene	Assay Nr	Symbol
Insulin	Rn01774648_g1	Ins2
Glucagon	Rn00562293_m1	Gcg
Somatostatin	Rn00561967_m1	Sst
Pancreatic polypeptide	Rn00561768_m1	Ppy
Ghrelin	Rn00572319_m1	Ghr1
Glucokinase	Rn00561265_m1	Gck
Hexokinase 1	Rn00562436_m1	HK1
Hexokinase 2	Rn00562457_m1	HK2
Hexokinase 3	Rn01448747_m1	HK3
Cyclophilin A	Rn00690933_m1	Ppia

## Supplementary information

Supplementary Information

Supplementary Information

## Data Availability

Raw data related to the single cell glucagon release experiments are provided as Supplementary Table S1. All other data are available on request from the authors.
