# Supplementary material for: Glucokinase intrinsically regulates glucose sensing and glucagon secretion in pancreatic alpha cells
Source: Sci Rep. 2020 Nov 19;10:20145. doi: 10.1038/s41598-020-76863-z (PMC7678872; doi:10.1038/s41598-020-76863-z)
Supplement: Supplementary file 1 — Supplementary Information [file 41598_2020_76863_MOESM1_ESM.pdf]

Supplementary Information

Glucokinase intrinsically regulates glucose sensing and glucagon secretion in pancreatic alpha cells

Tilo Moede, Barbara Leibiger, Pilar Vaca Sanchez, Elisabetta Daré, Martin Köhler, Thusitha P. Muhandiramlage, Ingo B. Leibiger* and Per-Olof Berggren*

Supplementary Methods

Culture and transfection of INS-1 cells

Rat insulinoma INS-1 cells [1] were cultured in RPMI 1640 medium supplemented with 2 mM glutamine, 10 mm HEPES (pH 7.4), 1 mm sodium pyruvate, 50 µM 2-mercaptoethanol and 10% FBS at 37 °C in a humidified atmosphere with 5 % CO_2.

To test the efficiency of the glucokinase siRNA the cells were transfected with siRNA against rat glucokinase (SI01515878) and validated non-targeting negative control siRNA (SI1027310) (both from QIAGEN, Hilden, Germany) using Lipofectamine 2000 (Thermo Fisher Scientific, Waltham, MA, USA). 5 days after start of transfection with the siRNA the cells were lysed and knockdown of glucokinase was confirmed by Western blot.

INS-1 cells were transfected with FITC-labelled non-silencing control siRNA for siRNA uptake assessment. The cells were transfected with the siRNA using Lipofectamine 2000 and imaged 30 h after start of transfection with an inverted laser scanning confocal microscope (TCS SP2; Leica Microsystems). FITC fluorescence was excited at 488 nm, and the fluorescence was detected at 505–535 nm. Efficiency of siRNA uptake was assessed by counting the percentage of transfected (green) cells relative to the total number of cells.

Western blot

INS-1 cells were washed with PBS and lysed in lysis buffer (50 mM Tris (pH 7.5), 1 mM EDTA, 1 mM EGTA, 0.5 mM Na_3VO_4, 0.1% (v/v) 2- mercaptoethanol, 1% Triton X-100, 50 mM NaF, 5 nM sodium pyrophosphate, 10 mM sodium β-glycerol phosphate, 0.1 mM PMSF, 1 µg/ml of aprotinin, pepstatine, leupeptin). The proteins (70 µg) were separated over a 10% SDS-polyacrylamide gel (buffering system according to Laemmli) and electrotransferred to PVDF membrane. The membrane was cut and probed with rabbit polyclonal glucokinase[2,3] antibody and mouse monoclonal GAPDH (clone 6C5[4]) antibody (both from Abcam, Cambridge, UK). Immunoreactivity was detected with horseradish peroxidase-conjugated secondary antibodies using the ECL system (Amersham, Piscataway, NJ, USA) and X-ray films (Fuji Photo Film Co., LTD., Tokyo, Japan). The films were scanned and the bands were quantified with Gelquant.NET (BiochemLabSolutions.com).

Supplementary References

1. Asfari, M. *et al.* Establishment of 2-mercaptoethanol-dependent differentiated insulin-secreting cell lines. *Endocrinology* **130**, 167–178 (1992).
2. Yan, X. *et al.* Control of hepatic gluconeogenesis by Argonaute2. *Mol. Metab.* **18**, 15–24 (2018).
3. Leibiger, B. *et al.* Insulin-feedback via PI3K-C2α activated PKBα/Akt1 is required for glucose-stimulated insulin secretion. *FASEB J.* **24**, (2010).
4. Anti-GAPDH antibody [6C5] - Loading Control (ab8245) | Abcam. Available at: https://www.abcam.com/gapdh-antibody-6c5-loading-control-ab8245.html. (Accessed: 7th October 2020)

Supplementary Figure 1

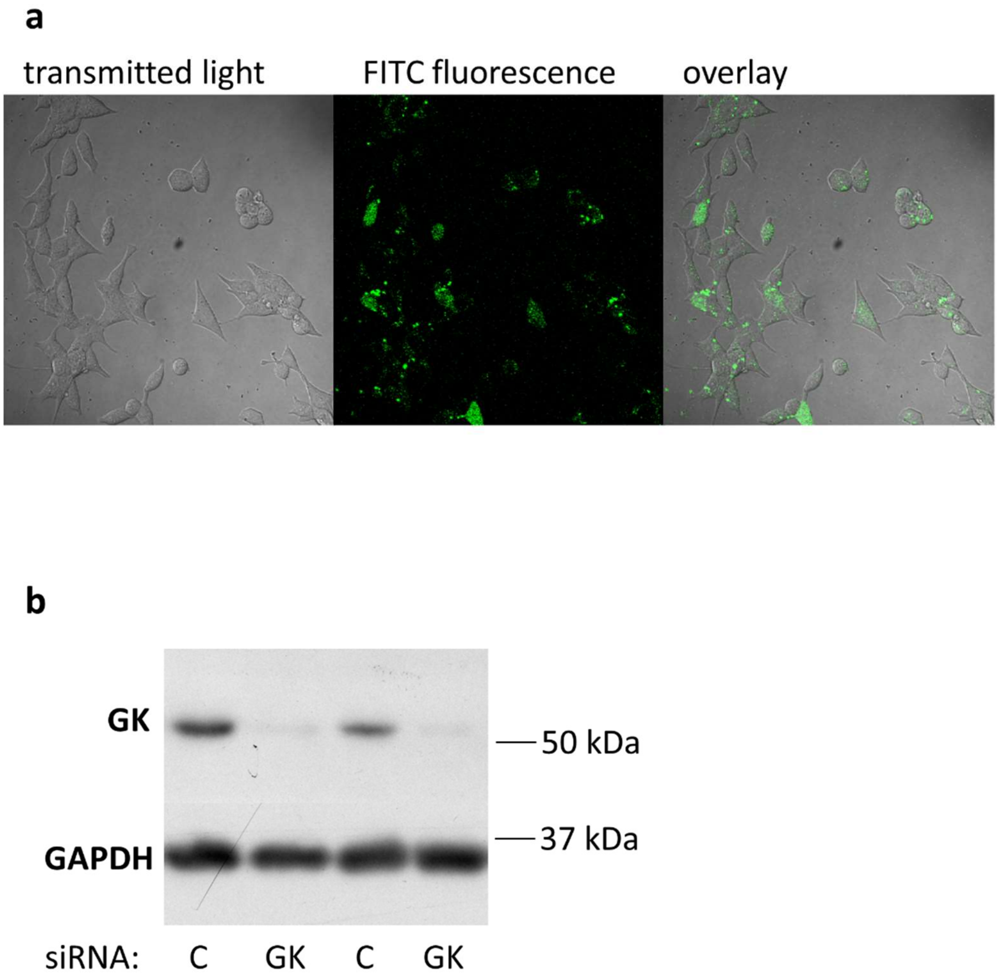

c

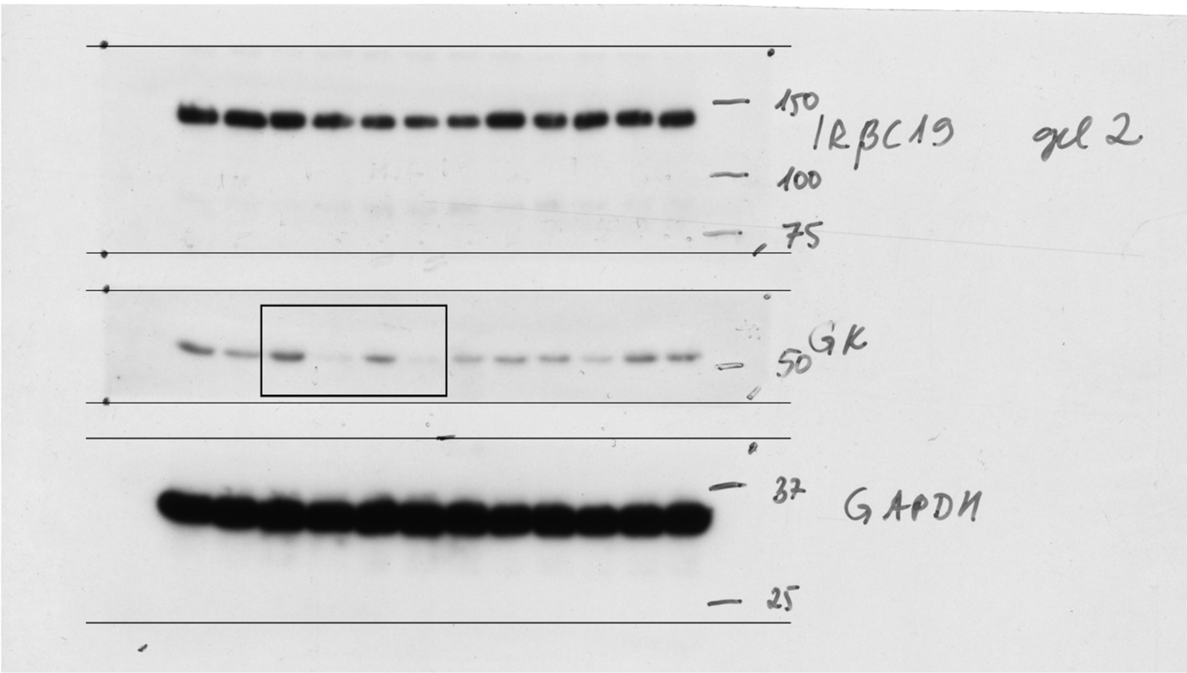

d

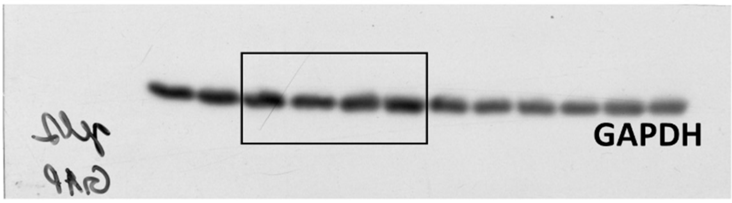

Supplementary Figure 1:

a) siRNA uptake in live INS-1 cells using fluorescence microscopy. Image of INS-1 cells transfected with FITC-labelled non-silencing control siRNA. Efficiency of siRNA uptake was about 90% (n=6). **b) Western blot of siRNA-transfected INS-1 cells.** Cells were transfected with non-silencing control (C) or glucokinase siRNA (GK) and the membrane was probed with glucokinase and GAPDH antibodies. Knockdown of glucokinase protein achieved was about 80% (n=2). **c), d) Original images of the Western blot shown in b).** The parts shown in b) are highlighted in c) GK and d) GAPDH. **c)** Original image of the Western blot that shows one exposure time of all parts of the cut membrane, lines indicate the borders of the membrane pieces. **d)** Original image of the GAPDH part of the blot that was exposed for a shorter time.
